# A Molecular Dynamics Simulation of the Human Lysozyme – Camelid VHH HL6 Antibody System

**DOI:** 10.3390/ijms10041719

**Published:** 2009-04-17

**Authors:** Zhi-Yuan Su, Yeng-Tseng Wang

**Affiliations:** 1 Department of Information Management, Chia Nan University of Pharmacy & Science, Tainan 717, Taiwan; 2 National Center for High-performance Computing, Tainan 742, Taiwan; E-Mail: c00jsw00@nchc.org.tw (Y.-T.W.)

**Keywords:** Molecular dynamics, amyloid diseases, lysozyme, atomic force microscopy

## Abstract

Amyloid diseases such as Alzheimer’s and thrombosis are characterized by an aberrant assembly of specific proteins or protein fragments into fibrils and plaques that are deposited in various tissues and organs. The single-domain fragment of a camelid antibody was reported to be able to combat against wild-type human lysozyme for inhibiting *in-vitro* aggregations of the amyloidogenic variant (D67H). The present study is aimed at elucidating the unbinding mechanics between the D67H lysozyme and VHH HL6 antibody fragment by using steered molecular dynamics (SMD) simulations on a nanosecond scale with different pulling velocities. The results of the simulation indicated that stretching forces of more than two nano Newton (nN) were required to dissociate the proteinantibody system, and the hydrogen bond dissociation pathways were computed.

## 1. Introduction

Alzheimer’s, an amyloid disease [1], was first identified in 1906 by Alois Alzheimer, a German neurological scientist. He observed plaques and neurofibrillary tangles in the pathological anatomical image of the brain of a female patient, and found that these would block the communications and the signal transmissions between nerves, a major cause of the progressive memory loss associated with the disease. The cause of the formation of neurofibrillary tangles is still unclear, while the formation of plaque is known to be induced by specific proteins, such as mutated lysozymes, aggregating with each other followed by their precipitation to form plaques in the brain. In 2003, Dumoulin [2] demonstrated *in vitro* that the Camelid VHH HL6 monoclonal antibody was able to effectively inhibit the aggregation of mutated lysozymes (I56T, F57I, W74R, and D67H) that would lead to the formation of fibrils or plaques and then cause amyloid diseases. The Camelid VHH HL6 antibody is the heavy chain of the antibody molecule, which shows particularly significant inhibition for the mutated lysozymes D67H and I56T The cAb-HuL6 antibody is a fragment of heavy-chain camel antibody with high specificity for human lysozyme, and the details of the three dimensional structure of the lysozyme-antibody complex can be found in Dumoulin *et al.* [3].

With regard to the simulation of amyloids, Nussinov’s group has done a lot of research on topics such as the short peptide amyloid organization [4] and the amyloid structural formation and assembly [5]. However, the present study focuses on steered molecular dynamics (SMD) simulations on model systems of lysozyme-antibody complex structures on c-terminal end-to-end extensions. Steered molecular dynamics was first introduced by Grubmuller [6] in 1996, and is a way to imitate the use of an atomic force microscope to detect the mutual interaction between two objects. SMD induces unbinding of ligands and conformational changes in biomolecules on time scales accessible to molecular dynamics simulations. Time-dependent external forces are applied to a system, and the responses of the system are analyzed. SMD has already provided important qualitative insights into biologically relevant problems, as demonstrated by various applications ranging from identification of ligand binding [7] and protein-protein interaction pathways [8] to explanation of the elastic properties of proteins. Detailed analysis of the SMD simulations on model systems of lysozyme-antibody complex structures reveals the range of the alteration of lysozyme-antibody hydrogen bond numbers, which are the pulling forces in the SMD extensions process.

## 2. Material and Methods

**Figure 1.**
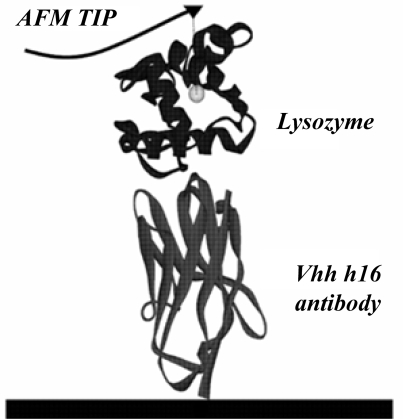
Schematic model of the steered molecular dynamics simulation.

The present study used the X-ray structure (PDB ID: 1op9) of the lysozyme–antibody complex published in the Protein Data Bank by Dumoulin [2] as the initial model. The antibody is a protein composed of 121 amino acids, while lysozyme is a protein made of 130 amino acids. The detailed calculation model is given in Figure 1.

Calculations were performed with the NAMD [9] and CHARMM [10] programs using the CHARMM27 all-hydrogen amino acid parameters [10]. The initial structure of the lysozyme-antibody was overlaid with a pre-equilibrated solvent box of the TIP3P water model (the size of the solvent box size was 15.4 × 13.5 × 7.5 nm^3^) and chorine ions. All water molecules within 0.19 nm of lysozymeantibody atoms were deleted and chorine ions added at random positions in the box in order to render the system electrostatically neutral. The size of the simulation system was 15.4 × 13.5 × 7.5 nm^3^, and it included 48,183 TIP3P water molecules. All MD simulations were performed in the isobaric, isothermal ensemble [11] with the simulation temperature was equal to 310 K, unless noted, using the verlet integrator, an integration time step of 0.002 ps and SHAKE [12] of all covalent bonds involving hydrogen atoms. In electrostatic interactions, atom-based truncation was undertaken individually using the PME method. The complex structures were minimized for 10,000 conjugate gradient steps. The minimized complex structures were then subjected to a 0.6 ns isothermal, constant volume MD simulation. The final structures from these simulations were then used to initiate the SMD calculations.

Steered molecular dynamics is based on the traditional molecular dynamics with the harmonic potential added on the atom or its aggregation. The complete harmonic potential function is illustrated below:

(1) $$U_{\text{harmonic}}\;=\;\frac{1}{2}\;K_{h}{\left(\vec{\nu }\times t-\vec{r}\right)}^{2}$$

where *K*_h_ represents the force constant of the harmonic potential function; $\vec{\nu }$ represents the pulling velocity of a virtual atom; *t* and *r* represent the simulation time and the coordinate of the atom or its aggregation with an additional action on itself. For the SMD simulation settings, the CA atom of the 121^st^ amino acid of the antibody was fixed first as a reference point. The additional harmonic potential function was then added to the CA atom of the 130^th^ amino acid of lysozyme with the force constant *K*_h_ of 4.32 kcal/(mol Å^2^). The 6 ns NVT ensemble simulation was conducted independently at pulling velocities of 0.00005, 0.00009, 0.00015, 0.00030, and 0.00090 Å per time-step.

## 3. Results and Discussion

**Figure 2.**
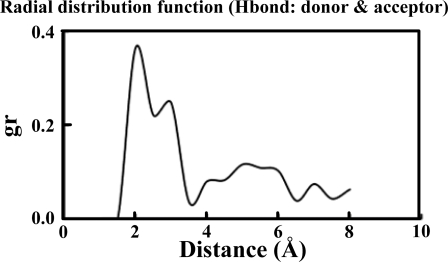
The profile of hydrogen bond RDF in the lysozyme-antibody complex simulation system.

**Figure 3.**
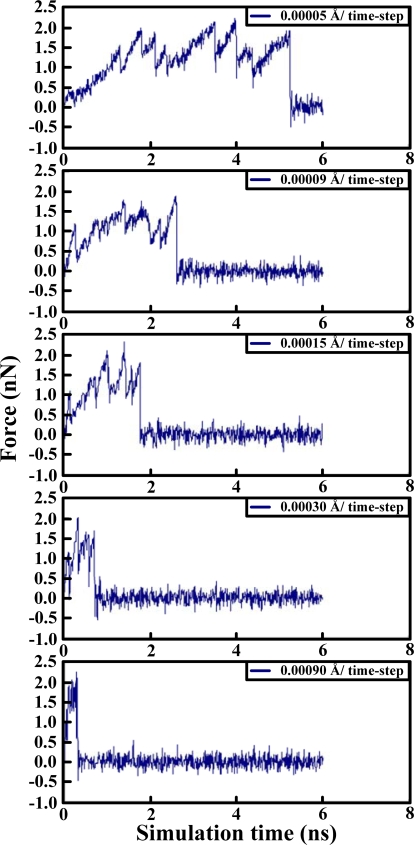
Corresponding force curves of pulling rates: 0.00005, 0.00009, 0.00015, 0.00030, 0.00090 Å/time-step.

**Figure 4.**
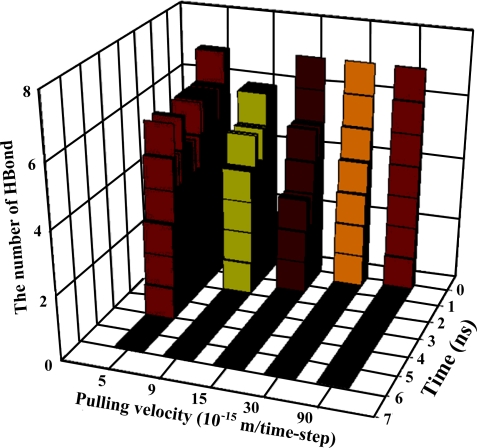
Calculating the amount of inter-molecular hydrogen bonds from pulling rates: 0.00005, 0.00009, 0.00015, 0.00030, 0.00090 Å/time-step.

**Figure 5.**
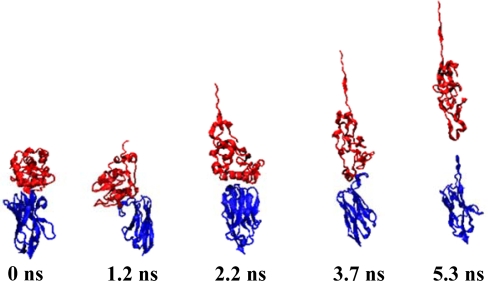
Snapshots from pulling rates: 0.00005 Å/time-step. Lysozye (red) and VHH HL6 antibody (blue).

Table 1 illustrates the atom types of the hydrogen bond donors and acceptors of the CHARMM force field, showing the results of the analysis of the radial distribution function (RDF) of the hydrogen bond donors-acceptors between two protein molecules. As shown in Figure 2, two strong hydrogen bonds were found at 2.2 and 2.4 Å, indicating the existence of such bonds between the proteins. The atoms of hydrogen bond formed are listed in Table 2. The results of the steered molecular dynamics simulation are shown in Figure 3, which also provides the strengths of forces required to dissociate two proteins under various pulling velocities. Using the data from Table 2, the number of existing hydrogen bonds (with a distance between the hydrogen bond donor and acceptor of less than 0.3 nm) under different pulling velocities was analyzed, and the results are shown in Figure 4, while the Snapshots (pulling rates: 0.00005 Å/time-step) are shown in Figure 5.

At the pulling velocity of 0.00005 Å per time-step, Figures 3 and 4 and Table 3 show that there were four peaks whose value was near 2 nN at the simulation times of 2 (HB ID: 3, 5, 6), 3.2 (HB ID: 3, 5, 6), 4 (HB ID: 3), and 5 (HB ID: Null) ns. The first three peaks caused the sudden decline in the number of hydrogen bonds between the two proteins, while the fourth peak induced the breakdown of the remaining van der Waals and Coulombic interactions between the two proteins. At the pulling velocity of 0.00009 Å per time-step, there were two major peaks at 1.8 (HB ID: 3, 5) and 2.7 (HB ID: Null) ns, with the first peak responsible for the disruption of the hydrogen bonds between two proteins and the second peak for the breakdown of the van der Waals and Coulombic interactions between the two. At the pulling velocity of 0.00015 Å per time-step, there were three major peaks at 0.9 (HB ID: 1, 3, 4, 5, 6), 1.5 (HB ID: 3, 5, 6), and 1.8 (HB ID: Null) ns, with the first two peaks as the major forces to disrupt the hydrogen bonds and the last one disrupting the non-contact interactions. At the pulling velocities of 0.00030 and 0.00090 Å per time-step, there are two peaks, at 0.2 (HB ID: 1, 3, 4, 5, 6) and 0.5 (HB ID: Null) ns, causing the interruption of non-contact and hydrogen bonding interactions between two proteins simultaneously.

Our simulation results suggest that the pulling velocities required at least 2 nN interactions to dissociate the lysozyme-antibody complex system and the unfolding pathway of two proteins could be clearly observed at the pulling velocity of 0.00005 Å per time-step. When the antibody was close to the lyszyme protein, the five hydrogen bonds (HB ID: 1, 3, 4, 5, 6) may play important roles in increasing the binding affinities of the complex system and make the antibody-lysozyme bind together more easily. After the binding of the antibody-lysozyme, the three hydrogen bonds (HB ID: 2, 7, 8) were still a key reason why the system remained stable.

## 4. Conclusions

The present study used steered molecular dynamics (SMD) to simulate the interactions between lysozyme and the Camelid VHH HL6 antibody. Our results show that the interaction of approximately 2 nN between two proteins and the eight hydrogen bonds may play important roles in increasing the binding affinities of the complex system and causing the antibody-lysozyme to bind together easily. However, the results should be treated with caution. With regard to protein dynamics, it is usually necessary to study an equilibrium ensemble of conformation; however, because we investigated a single equilibrated initial condition set in this study, the results are likely missing physically relevant entropic contributions. Furthermore, theoretically, it is very difficult for molecular dynamics simulations to use the fast pulling speeds used in this study. This is because there may be microstates which exist in the equilibrium ensemble where the hydrogen bonding network changes significantly in the large complex, and both members of the complex are then likely to undergo substantial conformational fluctuations, which are possibly coupled in a complex behavior. Therefore, a simulation just using a single initial conformation can not capture this complex phenomenon, and thus may underestimate the rupture force.

It should also be noted that the simulations used in this study required a large amount of time. Due to limited computational resources of our organization, all simulations were performed with only a 32-node (AMD Opteron 248 2.2GHz) PC cluster, and each SMD simulation (6 ns) case needed more than two months to be calculated. However, we think we have presented a meaningful preliminary test in this study, and building on this base, we would like to continue with this research in order to confirm the hydrogen bond dissociation pathway.

## Figures and Tables

**Table 1.** The atom types of the hydrogen bonds (CHARMM force field).

Hydrogen Bond	Atom type (Acceptor / Donor)
Acceptor	O, OD1, OD2, OE1, OE2, OG, OH, NE2, ND1
Donor	HN, HE, HE1, HE2, HH, HH11, HG1, HD1, HH12, HH21, HH22, HD21, HD22, HE21, HE22, HZ1, HZ2, HZ3

**Table 2.** The atoms of hydrogen bond formation.

A chain (antibody)		B chain (lysozyme)	HB (ID)
number of atom		simplified term	1

1423	3254	A1423:B3254	2
1604	3330	A1604:B3330	3
1603	3330	A1603:B3330	4
1603	3333	A1603:B3333	5
1554	3334	A1554:B3334	6
0727	3175	A0727:B3175	7
1444	3124	A1444:B3124	8

**Table 3.** The hydrogen bonds trajectories and simulations.

Pulling velocity (Å per time-step)	Simulations time (ns)	Existing hydrogen bonds (HB (ID))
0.00005	2	3, 5, 6
	3.2	3, 5, 6
	4	3
	5	Null
0.00009	1.8	3, 5
	2.7	Null
0.00015	0.9	1, 3, 4 ,5, 6
	1.5	3, 5, 6
	1.8	Null
0.00030	0.2	1, 3, 4 ,5, 6
	0.5	3, 5, 6
0.00090	0.2	1, 3, 4 ,5, 6
	0.5	3, 5, 6
